# To the Editors of the Medical and Physical Journal

**Published:** 1807-11

**Authors:** Robert Percival


### *To the Editors of the Medical and Physical Journal.*

GENTLEMEN,

HAVING seen my name quoted in your Journal, (p. 557, No. xciv.) as authority for the virtues of the roots of the Geum rivale, I beg leave to inform you, that I never made any trial of that plant. The roots of the Geum urbanum, in decoction, mixed with milk, I have known servicable in fluxes, and their powder, I believe, will be found at least as efficacious as that of many specimens of Peruvian bark, so called, which are to be met with in this country. These remarks I recollect to have made to some of my medical friends, who (with the mistake of the species) may have furnished them to the ingenious Compiler of your Indigenous Botany; a work, to which I should be happy to contribute any thing which might be worth communicating. Similar observations on the virtues of the roots of Geum urbanum, I need not inform him, occur in many writers. The roots afford a pleasant tincture.

I am, &c.

ROBERT PERCIVAL.

